# Cocaine Causes Apoptotic Death in Rat Mesencephalon and Striatum Primary Cultures

**DOI:** 10.1155/2015/750752

**Published:** 2015-07-29

**Authors:** Lucilia B. Lepsch, Cleopatra S. Planeta, Critoforo Scavone

**Affiliations:** ^1^Department of Pharmacology, Institute of Biomedical Sciences, University of São Paulo, Room 338, Avenida Professor Lineu Prestes 1524, 05508-900 São Paulo, SP, Brazil; ^2^LIBBS Company, São Paulo, SP, Brazil; ^3^Laboratório de Neuropsicofarmacologia, Faculdade de Ciências Farmacêuticas, Universidade Estadual Paulista, Araraquara, SP, Brazil

## Abstract

To study cocaine's toxic effects* in vitro*, we have used primary mesencephalic and striatal cultures from rat embryonic brain. Treatment with cocaine causes a dramatic increase in DNA fragmentation in both primary cultures. The toxicity induced by cocaine was paralleled with a concomitant decrease in the microtubule associated protein 2 (MAP2) and/or neuronal nucleus protein (NeuN) staining. We also observed in both cultures that the cell death caused by cocaine was induced by an apoptotic mechanism, confirmed by TUNEL assay. Therefore, the present paper shows that cocaine causes apoptotic cell death and inhibition of the neurite prolongation in striatal and mesencephalic cell culture. These data suggest that if similar neuronal damage could be produced in the developing human brain, it could account for the qualitative or quantitative defects in neuronal pathways that cause a major handicap in brain function following prenatal exposure to cocaine.

## 1. Introduction

Drug abuse can have physiological, psychological, and social consequences [1]. Cocaine is a drug of abuse with reinforcing proprieties that can lead to the development of dependence. By binding to plasma membrane transporters, cocaine prevents the uptake of extracellular monoamines, consequently enhancing their extracellular levels, including norepinephrine and dopamine [2–4].

The vast majority of developmental studies investigating cocaine effects have focused on the dopaminergic system, presumably as a result of dopamine's well-studied effects on reward and addiction [5]. The primary mesencephalic culture contains dopaminergic neurons from both the substantia nigra and ventral tegmental area, which expresses tyrosine hydroxylase (TH) [6, 7], the rate-limiting enzyme in dopamine synthesis. Dopaminergic afferents from substantia nigra pars compact provide dense innervations to the striatum [8, 9]. Given the reinforcing properties of cocaine such mesencephalic structures have been extensively investigated.

Besides its reinforcing properties, cocaine can cause damage to the CNS [10], being associated with cerebrovascular pathologies and convulsions that on occasion may be lethal [11]. More subtle functional and physical impairments may also be evident. Clinical and preclinical studies show learning and memory impairments, as well as the presence of movement disorders, following cocaine abuse, even after long periods of drug withdrawal [12, 13].

Cocaine can cross the placenta and accumulate in the fetus [14], with cocaine effects being especially evident in the newborns of females that abused cocaine during pregnancy. Maternal cocaine use during pregnancy is associated with significant impairment of cognitive development [15–17] that is detectable during the first two years of life and which may continue to contribute to learning difficulties and attentional dysfunction during later childhood [18]. In addition to the direct effects of cocaine, cocaine has a number of metabolites, which will be present in the mother and fetus and which have a number of biological effects, including local anesthesia [19] and the inhibition of monoamine transporters [20], as well as vascular effects [21] and seizure induction [22].

Prior work on the effects of gestational cocaine has shown apoptosis in the fetal heart [23], decreased birth weight and head size, and deficits in cognition, attention, and language development in childhood [24, 25]. The prenatal cocaine exposure can result in molecular adaptations or anatomy changes in specific brains regions, including the hippocampus and cortex [26, 27]. The mechanisms underlying the damage caused by cocaine may involve a number of factors, including mitochondrial dysfunction, toxicity from dopamine metabolism, and/or reactive oxygen species (ROS) formation [28]. The nature of any subsequent cell death may be via either apoptotic or necrotic cell death processes.

The aim of this study was to determinate the toxicity of cocaine in two different types of primary culture, striatal and mesencephalic. To our knowledge, this is the first study showing cocaine to cause cell death in such cultures. It is of note that the cocaine concentrations in this study are comparable to those of previous investigators, although in different cell types [29–32], as well as in the plasma of human drug abusers, ranging between 0.3 *μ*M and 1 mM [30].

## 2. Materials and Methods

### 2.1. Primary Mesencephalic/Striatal Cultures

Primary cultures were cultured as previously described [33]. In brief, the mesencephalon or striatum of Sprague-Dawley rat embryos on embryonic day 17 was isolated and digested with 0.5 mg/mL trypsin in Earle's Balanced Salt Solution (EBSS) (Life Scientific) for 2 hr at 37°C with 5% CO_2_ and plated on poly-L-lysine (Sigma) coated glass coverslips on plastic culture dishes (MatTek), at a density of  1 × 10^6^ cells/mL in high glucose Dulbecco's minimum essential medium (DMEM) supplemented with 10% bovine calf serum, 25 U/mL penicillin, 25 mg/mL streptomycin, and 2 mM glutamine (Invitrogen). These mixed neuronal/glial cultures were treated with cocaine hydrochloride (Sigma) 1.0 mM or phosphate saline buffer (PBS) as control, on day 9* in vitro*, for 24 hours. The chosen cocaine concentration was based on previous studies [34].

### 2.2. Immunostaining

On day 10, after 24 hours* in vitro*, neurons were identified by staining with anti-MAP2 (1 : 100; Sigma) or anti-NeuN (1 : 100; Chemicon; MAB 377) [33, 35]. Unless otherwise stated, each experiment described below was repeated at least three times, and >100 neurons were scored for each condition on triplicate coverslips. After 24 hours of cocaine exposure, cultures were fixed with 4% paraformaldehyde (Sigma) in PBS and permeabilized with 0.1% Triton X-100. After blocking nonspecific binding with PBS plus 3% BSA and 3% fetal bovine serum, the cells were incubated with antibodies to identify neurons (anti-MAP2 or anti-NeuN) followed by secondary Alexa Fluor 594 goat anti-mouse antibodies (1 : 100; Molecular Probes). In the last wash step, Hoechst 33324 (1 g/mL) was added to assess nuclear morphology. Hoechst 33342 is a UV-excitable nucleic acid stain readily taken up by all cells. Its blue fluorescence is particularly bright in the condensed nuclei of apoptotic cells. Typically, several hundred cells were scored in each experiment using fluorescent microscopy.

### 2.3. TUNEL Assay

Cells with DNA fragmentation were detected by the terminal deoxynucleotidyl transferase-mediated biotinylated UTP nick end labeling (TUNEL) method using the “*in situ* cell death detection-fluorescein kit” (Roche).

### 2.4. Statistical Analysis

Data were obtained from three independent experiments. In each experiment three replicate samples were quantified. Statistical comparisons were made by Student's* t-test* for single comparisons. All values of *P* < 0.05 were considered statistically significant.

## 3. Results

To characterize the primary striatum culture, on day 10* in vitro*, we observed the expression of GABAergic neurons that were stained with anti-GAD65/67 [36]. Our results demonstrated that 90% of the neurons present in the culture were GABAergic neurons (data not shown). We also tested for the presence of dopaminergic neurons by antibody staining to identify tyrosine hydroxylase (TH), the rate-limiting enzyme in the dopamine synthetic pathway. The results showed that there was no sign of striatal neurons expressing TH (data not shown). The mesencephalic culture was positive for TH, indicating that our mesencephalic culture comprises 10% dopaminergic neurons, which is characteristic of mesencephalic cultures [6].

**Figure 1:**
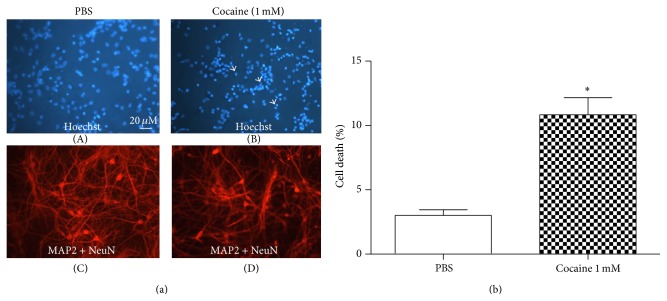
(a) Immunostaining of striatal primary cells treated with PBS (left panel) or treated with cocaine 1.0 mM (right panel) for 24 hours. Neurons were labeled with MAP2 and NeuN (red label). Hoechst 33342 (blue label) was added to monitor chromatin condensation. Arrows indicate dying neurons. Staining was observed under a fluorescent microscope. The treatment with cocaine caused a decrease in neuronal viability and an inhibition of neurite prolongation. (b) Percentage of cell death observed by immunostaining of striatal primary cultures treated with PBS and cocaine for 24 hours. Values are mean ± SEM from five independent experiments. The treatment with cocaine caused a decrease in the viability of the neurons. ^*∗*^Significantly different from the control (PBS) value: ^*∗*^ *P* < 0.05 by Student's* t-test.*

In the primary striatal culture, control neurons exhibited normal chromatin, showing only 3% cell death. In contrast, after cocaine treatment (1.0 mM, 24 hours), neurons manifested an increase in bright/condensed Hoechst 33342 fluorescence, with evidence of 10% cell death (Figures 1(a) and 1(b)). We also observed, as indicated by MAP2 and NeuN staining, that neurite extension was inhibited after cocaine treatment (Figure 1(a)).

**Figure 2:**
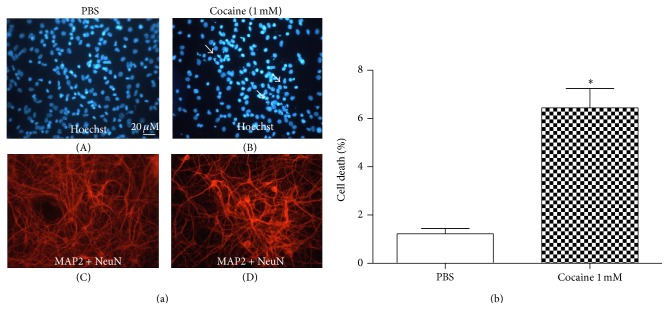
(a) Immunostaining of mesencephalic primary cells treated with PBS (left panel) or treated with cocaine 1.0 mM (right panel) for 24 hours. The neurons were labeled with MAP2 and NeuN (red labels). Hoechst 33342 (blue label) was added to monitor chromatin condensation. Arrows indicate dying neurons. Staining was observed under a fluorescent microscope. Cocaine treatment caused a decrease in neuronal viability. (b) Percentage of cell death observed by immunostaining of the mesencephalic primary culture treated with PBS and cocaine for 24 hours. Values are mean ± SEM from four independent experiments. Cocaine treatment decreased neuronal viability. ^*∗*^Significantly different from the control (PBS) value: ^*∗*^ *P* < 0.05 by Student's* t*-*test*.

Similarly, in the primary mesencephalic culture, treatment with cocaine (1.0 mM) for 24 hours caused a decrease in neuronal viability coupled to an inhibition of neurite prolongation (Figures 2(a) and 2(b)).

**Figure 3:**
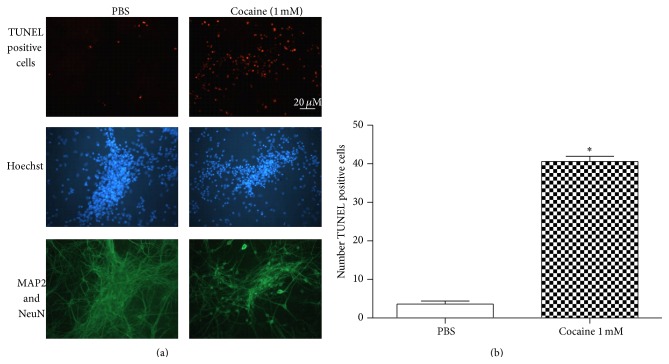
(a) *In situ* histochemical evidence of DNA fragmentation after cocaine exposure. Striatal cultures were first established for 7 days and incubated with cocaine (1.0 mM) for 24 hours. After cells were fixed, the TUNEL method was performed. Cultures were photographed at the level of the neuronal layer. Note the labeling in the vast majority of treated cells, in contrast with the labeling of a few control cells. TUNEL positive cells were dUTP labeled (brown label). The neurons were labeled with MAP2 and NeuN (green label) and Hoechst 33342 (blue label) was added to monitor chromatin condensation. (b) Number of TUNEL positive cells observed by immunostaining of the striatal primary culture treated with PBS or cocaine for 24 hours. Values are mean ± SEM from five independent experiments. Cocaine treatment decreased neuronal viability. ^*∗*^Significantly different from the control (PBS) value: ^*∗*^ *P* < 0.05 by Student's* t*-*test*.

**Figure 4:**
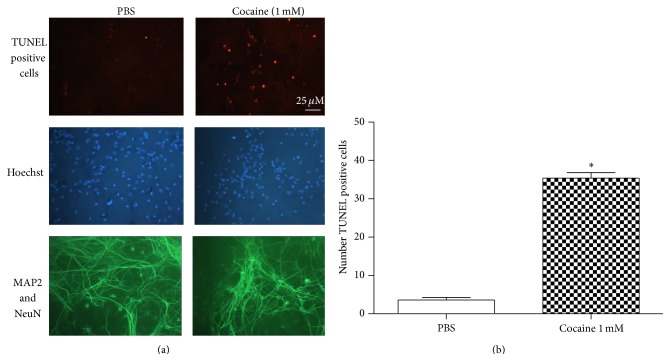
(a) Mesencephalic cultures were first established for 7 days and incubated with cocaine (1.0 mM) for 24 hours. After cells were fixed, the TUNEL method was used. Cultures were photographed at the level of the neuronal layer. Note the labeling in the vast majority of treated cells, in contrast with the labeling of a few control cells. TUNEL positive cells were dUTP labeled (brown label). The neurons were labeled with MAP2 and NeuN (green label) and Hoechst 33342 (blue label) was added to monitor chromatin condensation. (b) Number of TUNEL positive cells observed by immunostaining of the mesencephalic primary culture treated with PBS or cocaine for 24 hours. Values are mean ± SEM from five independent experiments. Cocaine treatment decreased neuronal viability. ^*∗*^Significantly different from the control (PBS) value: ^*∗*^ *P* < 0.05 by Student's* t*-*test*.

The TUNEL assay confirmed that cocaine caused apoptotic death in both striatal and mesencephalic cultures (Figures 3(a), 3(b), 4(a), and 4(b)).

## 4. Discussion

Cocaine abuse can lead to toxic effects, including causing damage in specific brain areas. Studies in humans [37, 38], animals [39], and cell cultures [40, 41] have shown the toxic effects of cocaine, which can lead to cell death. Neuronal death during CNS development can change the organization of synaptic connectivity, leading to developmental and behavioral abnormalities in the offspring. Previous work shows cocaine to modulate the development [42–44] and survival [43–45] of CNS cells.

The present study demonstrates that cocaine decreases neuronal survival in primary striatal and mesencephalic cultures, two different brain regions relevant to cocaine's mechanism of action. Most neurons in striatal culture are GABAergic, with some cholinergic neurons. Also, striatal cultures of primary neurons express functional D_1_ and D_2_ dopamine receptors [46, 47] as well as the dopamine transporter [7]. We also observed morphological changes in both cultures, characterized by chromatin condensation and DNA fragmentation, which indicates a process of apoptosis. In our model, striatal neurons in cell culture do not express the TH enzyme, the rate-limiting enzyme in dopamine synthesis, suggesting that this culture cannot produce dopamine. However, mesencephalic neurons in culture did express TH and therefore produce dopamine. Given the cocaine toxicity in both cultures, this suggests that cocaine's toxic effect may be regulated by dopamine, but also possibly by an array of signaling through multiple and diverse secondary messenger system(s).

We showed that the exposure of both primary mesencephalic and striatal culture neurons to cocaine evoked an apoptotic process. Apoptosis has also been reported by some authors in other models but not always [48–50]. It might be that the great variability in the physiological and functional effects of cocaine on developing CNS is due to the multiple biochemical and pathophysiological routes of cocaine's actions. For example, dopamine and 5-hydroxytryptamine (serotonin) transporter knock-out mice still continue to exhibit drug seeking behavior, suggesting the involvement of additional molecular pathway for cocaine action, besides blocking monoamine neurotransmitter transporters [4]. Studies demonstrated that GABA transmission in the nucleus accumbens is also altered after withdrawal from repeated cocaine [51]. At higher concentrations, cocaine can act as a local anesthetic, interacting with a variety of targets in both specific and nonspecific manners.

Chronic cocaine treatment can modulate voltage-gated Na^+^ and Ca^2+^ channels activity via the production of cyclic AMP by DA_1_ receptor stimulation [52, 53]. Also, direct modulation of ion channels can be responsible for some cocaine effects. It has been shown that cocaine can block voltage-dependent Na^+^ channels [54] and Ca^2+^ channels [53]. Other ion channels are modified by cocaine including the K^+^ channels activated by acetylcholine and adenosine [55]. Ca^2+^ and K^+^ channels are involved in the repolarization and after-hyperpolarization phases of the action potential. The magnitude and duration of the after hyperpolarization phase determine the rate of neuronal firing. Blockade of the Ca^2+^-activated-K^+^ channels may facilitate repetitive neuronal firing that may enhance the propensity to induce seizures and neuronal function during cocaine overdose [56]. Blockade of K^+^ could also underline a variety of effects mediated by cocaine, including increased Ca^2+^ influx at the presynaptic terminal, which can augment neurotransmitter and hormone release and can contribute to neurodegenerative processes. As such ionic regulation may be a significant mediator of cocaine's neurotoxicity. Although we have no evidence of the presence of cocaine metabolites (ecgonine, ethyl ecgonine, and ecgonine methyl ester) in these cultures, we cannot rule out that they could also be involved in the mechanism of cell death. However, previous work showed that only cocaine significantly decreased MAP2 content in cortical culture [57]. This could suggest that the apoptotic cascade might require the intracellular penetration of cocaine.

We also observed an inhibition of the neurite outgrowth in the cells exposed to cocaine. This could be due to the influence of cocaine on cytoplasmic calcium, thereby affecting the cytoskeletal network and altering neuronal regulation. Cocaine may target cytoskeleton proteins, particularly microtubule associated proteins (MAPs) [58] and actin filaments, altering the process of initiation, elongation, and turning of neuritic branches [59]. Cocaine can also act to modulate integrin structure and functions, thereby contributing to decreased neurite outgrowth. Nonintegrin ligands can alter neuronal integrin expression, with consequences for neurite outgrowth [60].

Maternal gestational cocaine abuse can cause damage to their offspring. Since the migration of neurons ultimately determines their connectivity, synaptic potential, and viability, altered neuronal migration may be a significant determinant of the consequences of maternal gestational cocaine use in the offspring. Here we demonstrate, for the first time, that an acute dose of cocaine can cause the apoptosis of primary striatal and mesencephalic culture cells after 24 hours. Further investigation as to the biological underpinnings of cocaine's effects is likely to contribute to the etiology, course, and treatment of the consequences of maternal gestational cocaine abuse in the offspring.
